# Deciphering the Binding of the Nuclear Localization Sequence of Myc Protein to the Nuclear Carrier Importin α3

**DOI:** 10.3390/ijms232315333

**Published:** 2022-12-05

**Authors:** Bruno Rizzuti, Juan L. Iovanna, José L. Neira

**Affiliations:** 1 CNR-NANOTEC, SS Rende (CS), Department of Physics, University of Calabria, 87036 Rende, Italy; 2 Instituto de Biocomputación y Física de Sistemas Complejos–Unidad Mixta GBsC-CSIC-BIFI, Universidad de Zaragoza, 50018 Zaragoza, Spain; 3 Centre de Recherche en Cancérologie de Marseille (CRCM), INSERM U1068, CNRS UMR 7258, Institut Paoli-Calmettes, Aix-Marseille Université, 13288 Marseille, France; 4 Instituto de Investigación, Desarrollo e Innovación en Biotecnología Sanitaria de Elche (IDIBE), Universidad Miguel Hernández, 03202 Elche, Spain

**Keywords:** nuclear localization signal, binding, biolayer interferometry, calorimetry, fluorescence, molecular docking

## Abstract

The oncoprotein Myc is a transcription factor regulating global gene expression and modulating cell proliferation, apoptosis, and metabolism. Myc has a nuclear localization sequence (NLS) comprising residues Pro320 to Asp328, to allow for nuclear translocation. We designed a peptide comprising such region and the flanking residues (Ala310-Asn339), NLS-Myc, to study, in vitro and in silico, the ability to bind importin α3 (Impα3) and its truncated species (ΔImpα3) depleted of the importin binding domain (IBB), by using fluorescence, circular dichroism (CD), biolayer interferometry (BLI), nuclear magnetic resonance (NMR), and molecular simulations. NLS-Myc interacted with both importin species, with affinity constants of ~0.5 µM (for Impα3) and ~60 nM (for ΔImpα3), as measured by BLI. The molecular simulations predicted that the anchoring of NLS-Myc took place in the major binding site of Impα3 for the NLS of cargo proteins. Besides clarifying the conformational behavior of the isolated NLS of Myc in solution, our results identified some unique properties in the binding of this localization sequence to the nuclear carrier Impα3, such as a difference in the kinetics of its release mechanism depending on the presence or absence of the IBB domain.

## 1. Introduction

The oncogene *MYC* is estimated to contribute to 75% of all human cancers [1,2], many of which are aggressive and respond poorly to the current therapies [3]. Human c-Myc protein (from now on indicated as Myc) is a pleiotropic transcription factor encoded by the *MYC* gene, which is often amplified in cancer. Myc acts as a central hub inside the nucleus, modulating signals from several pathways to direct gene expression programs and control many biological functions, such as cell proliferation, growth, differentiation, apoptosis, and metabolism [4,5,6]. Under normal, non-stressing conditions, Myc is under tight genetic control in the cell, but defects in its regulation lead to overexpression in many cancers [7]. Its expression levels in the cell, and its activity, are controlled at both the transcriptional and protein level by multiple mechanisms, such as gene expression, negative autoregulation, and mRNA/protein stability and degradation, many of which are deregulated in human cancers [1]. Inhibition of Myc in vivo leads to sustained tumor regression due to promoting proliferative arrest, apoptosis, differentiation, and cellular senescence in cancer cells, while the anti-proliferative effects in healthy tissues are reversible and minimal [8].

**Figure 1:**
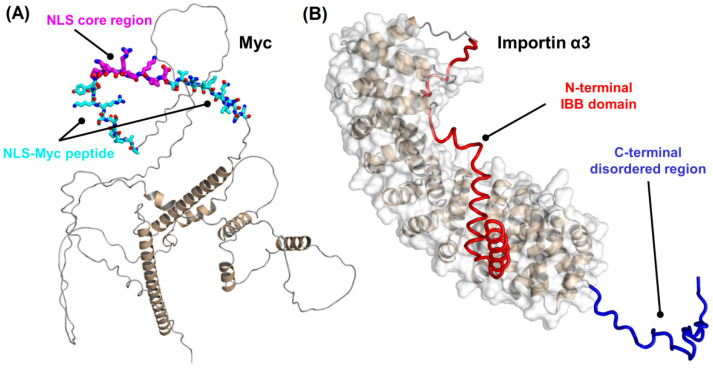
Structure of: (**A**) Myc; and (**B**) Impα3. Because of the high degree of disorder in large regions of both proteins, the conformations shown are those predicted by AlphaFold [14] and deposited in its Protein Structure Database (entries: P01106 and O00629, respectively). For Myc, the NLS-Myc peptide (cyan) includes the NLS core (magenta), and the side chains are explicitly shown. For Importin α3, the N-terminal IBB domain (red) is shown in its auto-inhibitory self-binding conformation, the volume of the highly ordered ARM-region is highlighted (white solid surface), and the disorder-prone C-terminal region (blue) is represented in extended conformation.

Human Myc has several conserved regions that are functionally important: a mainly disordered N-terminal transactivation domain, and a C-terminal region, comprising the basic, helix-loop-helix leucine zipper, dimerization and DNA-binding domains [7] (Figure 1A). The so-called canonical nuclear localization sequence (NLS) of Myc [9] lies at the C-terminal region of the protein, on residues 320–328, and it is mainly responsible for the transport of Myc into the nucleus through association with Importin α (and thus, it follows the classical nuclear transport route [10,11,12]; see next paragraph); in fact, the X-ray structure of the region comprising the NLS of Myc [9] in complex with importin α of *Saccharomyces cerevisiae* has been solved [13]. The canonical NLS includes a so-called core region encompassing residues 323–326, which in Myc has the sequence K^323^RVK^326^; this core region obeys to the general rule for monopartite NLS sequences binding to importin α, which have the sequence K(K/R)*X*(K/R) [10], where *X* represents any amino acid.

Nuclear translocation generally occurs through importins, together with other auxiliary proteins [11,12]. The classical nuclear import pathway is triggered by the recognition of an NLS polypeptide patch in the cargo by importin α [11]. The complex cargo-importin α then binds to importin β, and the so-formed ternary complex moves through the nuclear pore complex (NPC). Importin α is a modular protein (Figure 1B) with several α-helix repeat armadillo (ARM) units [11,15]. It is composed of two domains: (i) an N-terminal importin β-binding (IBB) domain, approximately 60-residue long, which is used for binding to importin β before the transport through the NPC; and (ii) a C-terminal NLS-binding motif formed by ten ARM units, which ends with the disorder-prone C-terminal region. When importin β is not present, then the IBB domain, which mimics an NLS, occupies the ARM regions implicated in NLS recognition. This intramolecular interaction has an auto-inhibitory role, and it is thought to be relevant in cargo dissociation in the nucleoplasmic side [15].

We considered importin α3 (Impα3), and its deletion mutant (ΔImpα3) depleted of the IBB domain, as targets for Myc based on several reasons. First, because of the larger flexibility of Impα3 compared with other importins, as concluded by the structural B-factors from X-ray data; this feature confers this isoform a greater ability to interact with various cargos [16] and, in fact, it is thought to be the reason behind its high conservation across different species [17]. Second, Impα3 can be considered a model protein to investigate how the NLS of the cargo can affect the thermodynamic parameters in the binding process, and we have already carried out several studies of the binding of Impα3 with other NLSs that we can use as a comparison [18,19,20]. Third, Impα3 has been recently used as a carrier of a modified peptide with a fluorescence probe, encompassing the NLS of Myc [21]. We also note that, from a more practical point of view, Impα3 can also be easily expressed and purified for in vitro structural and binding studies [16,17,18]. Moreover, by studying both species (with and without the IBB) of the same importin paralogue, we could explore whether the absence of the IBB domain affects the binding of NLS-Myc, as it has been studied in the case of other NLSs of several proteins (see [18,19,20], and references therein).

In recent years, we have been studying the conformational propensities of the NLSs belonging to several proteins (either fully folded or disordered), and their binding reactions to the same model importin (i.e., Impα3), to address several issues: First, to elucidate whether the NLSs are disordered when isolated in solution [19,20,22], no matter how the structure of the protein they belong to is, or by contrast whether they possess any structure. Second, to understand the relevant thermodynamic parameters (enthalpy and free energy) that govern their binding reaction [18,19,20], for assessing how these parameters are sequence-dependent. And finally, in the long term, after recognizing the driving interactions that govern the binding of several NLSs to a model importin, to design new drugs or small peptides hampering such binding, selectively inhibiting translocation at the cellular level to obtain a therapeutic effect [19,23].

In our experiments in the current study, we firstly observed that NLS-Myc was disordered in solution when isolated, as shown by NMR. Then, we explored the association between the peptide and either Impα3 or ΔImpα3, by using fluorescence, circular dichroism (CD) and biolayer interferometry (BLI). The combined used of these techniques confirmed that the binding took place, with dissociation constants in the low micromolar range (0.5 µM for Impα3, and 60 nM for ΔImpα3). Moreover, molecular docking simulations suggested that the core region of NLS-Myc was responsible for the binding, and that it was capable of anchoring to the major binding site of Impα3. The overall results were then discussed in the context of the analogies or differences in the binding to Impα3/ΔImpα3 of NLS-Myc when compared to other cargo peptides or proteins.

## 2. Results

### 2.1. Conformational Features of Isolated NLS-Myc

**Figure 2:**
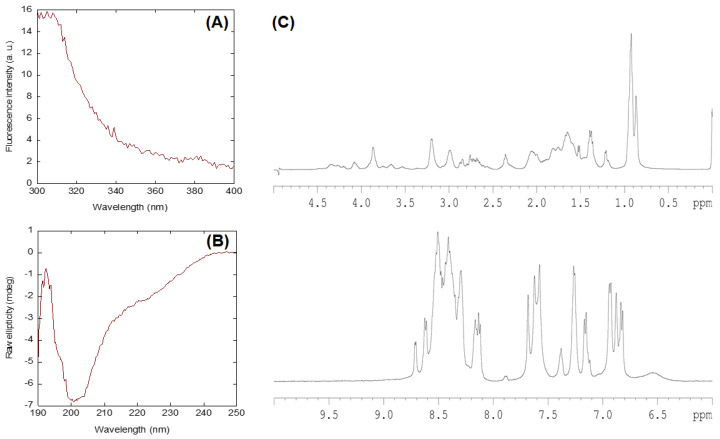
Conformational features of NLS-Myc: (**A**) fluorescence spectrum obtained by excitation at 280 nm of the isolated NLS-Myc. Experiments were carried out at 25 °C; (**B**) far-UV CD spectrum of the isolated NLS-Myc. Experiments were carried out at 25 °C; and (**C**) 1D ^1^H-NMR spectrum of the NLS-Myc showing the methyl (top) and the amide (bottom) regions. The signal at 0 ppm in the top section of the spectrum corresponds to TSP. Experiments were carried out at 10 °C.

We first studied the conformational features of the NLS-Myc peptide in isolation by using fluorescence, CD and NMR. The fluorescence spectrum was typical of a polypeptide with a sole tyrosine (Figure 2A) with a maximum at 308 nm. The far-UV CD spectrum corresponded to that of a random-coil polypeptide, with a minimum around 203 nm and a shoulder at ~225 nm (Figure 2B). It could be suspected that, due to the presence of two consecutive proline residues (Pro312 and Pro313 a third proline residue is also present later in the sequence, Pro320), the peptide might also show a small fraction of poly-proline II conformation; however, the far-UV CD spectrum (Figure 2B) lacked the positive band around 225 nm, which is a feature of this type of conformation [24]. Deconvolution of far-UV CD spectrum, by using the DICHROWEB site [25] and different deconvolution programs, yielded percentages of random-coil conformations of 47% or higher, while those of α-helix were always lower than 15%.

The disordered character of NLS-Myc was further confirmed by the 1D-^1^H-NMR spectrum (Figure 2C), which showed a clustering of signals of all the amide protons between 8.0 and 8.6 ppm, whereas the methyl protons were observed between 0.8 and 1.0 ppm. Furthermore, hydrogen-exchange experiments in the presence of D_2_O resulted in the disappearance of all the amide signals after 5 min of dissolving the sample, suggesting that none of the amide protons was hydrogen-bonded (Figure S1). To further confirm the mainly disordered nature of the peptide, we also carried out homonuclear 2D-^1^H-NMR experiments. We could not fully assign all the resonances due to the large number of Lys residues (Table S1). The peptide was mainly disordered in solution, as suggested by two lines of evidence. First, the sequence-corrected conformational shifts (Δδ) of H_α_ protons [26,27,28] for unambiguously assigned residues, far away from both terminal regions, were within the commonly accepted range for random-coil peptides (Δδ ≤ 0.1 ppm) (Table S1). Second, no long- or medium-range NOEs were detected, but only sequential ones (i.e., αN (*i*, *i* + 1) and βN (*i*, *i* + 1)) were observed in the polypeptide patches fully assigned (Figure S2). These results further confirm the findings from far-UV and 1D-^1^H-NMR spectra (Figure 2).

NLS-Myc was monomeric in solution, as concluded from the value of the diffusion coefficient *D* measured by the DOSY experiments (Figure S3), and from the estimated hydrodynamic radius *R*_h_ obtained from a comparison with the *D* of dioxane, which is (5.3 ± 0.6) × 10^−6^ cm^2^ s^−1^. In fact, the two values determined for the peptide were, respectively, *D* = (1.14 ± 0.08) × 10^−6^ cm^2^ s^−1^ and *R*_h_ = 10 ± 2 Å. The value of *R*_h_ was similar to that obtained theoretically for a random-coil polypeptide [29] with a corresponding molecular weight (3351.89.54 Da): 15 ± 2 Å. The small difference with the predicted value could be due to the constraints caused by the presence of the three Pro residues in the sequence, because of its relatively large fraction (10%) in this 30-residue-long peptide compared to most peptide sequences.

### 2.2. NLS-Myc Could Bind to Both Impα3 and ΔImpα3

To test whether NLS-Myc interacted with Impα3 and ΔImpα3 in vitro, we followed a two-part experimental approach. First, we used steady-state fluorescence and CD as spectroscopic techniques to observe a possible binding, and concomitant conformational changes in any of the three polypeptides; and secondly, we used fluorescence and BLI to quantitatively measure the thermodynamic parameters of such binding.

**Figure 3:**
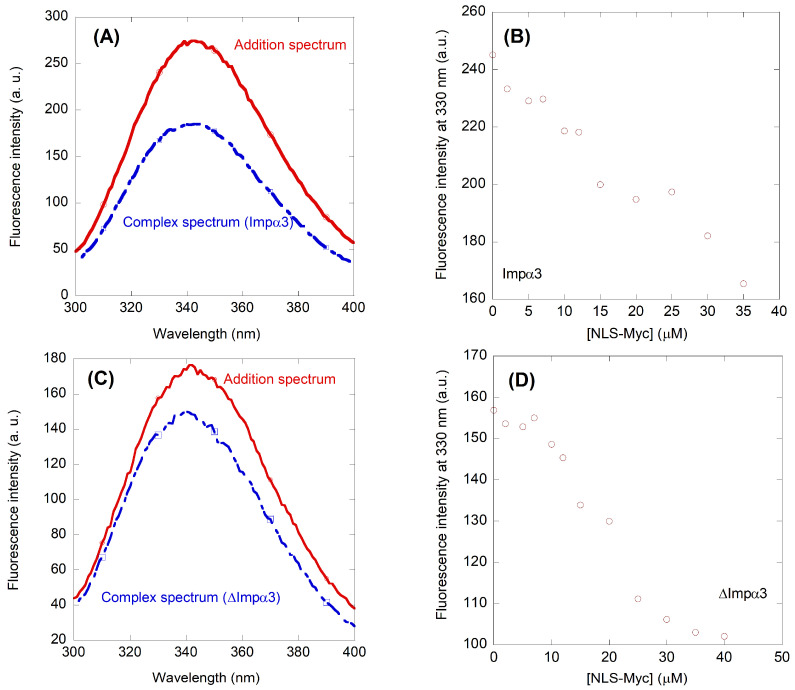
Binding of Impα3 and ΔImpα3 to NLS-Myc as monitored by fluorescence: (**A**) fluorescence spectrum obtained by excitation at 280 nm of the Impα3/NLS-Myc complex, and addition spectrum obtained by the sum of the spectra of the two isolated macromolecules; (**B**) titration curve monitoring the changes in the fluorescence at 330 nm when NLS-Myc was added to Impα3. The fluorescence intensity on the y-axis is the relative signal after removal of the corresponding blank; (**C**) fluorescence spectrum obtained by excitation at 280 nm of the ΔImpα3/NLS-Myc complex, and addition spectrum obtained by the sum of the spectra of the two isolated macromolecules; and (**D**) titration curve monitoring the changes in the fluorescence at 330 nm when NLS-Myc was added to ΔImpα3. The fluorescence intensity on the y-axis is the relative signal after removal of the corresponding blank. All experiments were carried out at 25 °C. Errors in panels (**B**,**D**) were estimated to be 10%, as judged by the repetitions of three titration experiments.

We used fluorescence to determine whether there was a change in: (i) the position of the maximum wavelength; (ii) the fluorescence intensity at that wavelength; or (iii) both of them, when the spectrum of the complex was compared to that obtained from the addition of the separated spectra of the two isolated polypeptides. A variation in fluorescence intensity by excitation at 280 nm was observed when the corresponding complex with either Impα3 or ΔImpα3 was formed (Figure 3A,C), but there were no changes in the maximum wavelength of the spectrum; similar variations were observed by excitation at 295 nm for the complex with either Impα3 or ΔImpα3. The fluorescence spectra of both isolated Impα3 and ΔImpα3 have been described previously [30], and they have maxima at 340 nm, as those of the complexes (Figure 3A,C). Because the spectra of isolated NLS-Myc have only a maximum at 308 nm (Figure 2A), the spectra of the complexes are dominated by the signal of each importin species.

**Figure 4:**
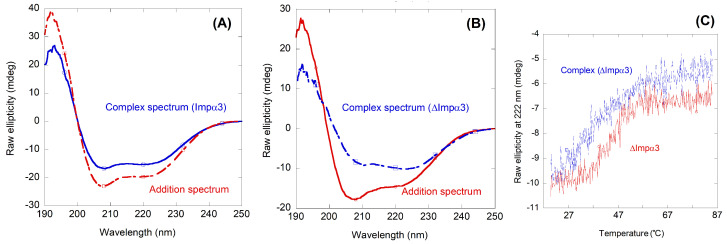
Binding of Impα3 and ΔImpα3 to NLS-Myc as monitored by far-UV CD: (**A**) far-UV CD spectrum of Impα3/NLS-Myc complex, and addition spectrum obtained by the sum of the spectra of the two isolated macromolecules; (**B**) far-UV CD spectrum of ΔImpα3/NLS-Myc complex, and addition spectrum obtained by the sum of the spectra of the two isolated macromolecules. All experiments were performed at 25 °C; and (**C**) thermal denaturations of the ΔImpα3/NLS-Myc complex and isolated ΔImpα3 were followed by the changes in ellipticity at 222 nm.

Next, we carried out far-UV CD measurements, trying to confirm the fluorescence binding results. The far-UV CD spectra of isolated Impα3 or ΔImpα3 have been described previously [30], and they showed the typical signal of an α-helix protein, with minima around 208 and 222 nm. As the signal of isolated NLS-Myc is not very intense (Figure 2B), the far-UV CD spectra of the complexes are dominated by those of each importin species. As in the fluorescence experiments, there were large differences between the addition spectra and those of the corresponding complexes of the peptide with either Impα3 or ΔImpα3 (Figure 4A,B). Since it is unlikely that the structures of any of the two importin species (which have ~500 amino acid residues) could change upon binding to NLS-Myc (a 30-residue-long peptide), the changes in the far-UV CD spectra must be due to: (i) changes in the polar environment of some of the aromatic residues of the importin species (as NLS-Myc has only a tyrosine residue); and/or (ii) changes in the secondary structure of the peptide upon binding. Since the X-ray structure of the complex between importin α of *Saccharomyces cerevisiae* and the core region of NLS-Myc has been solved [13], and there are no large changes in the secondary structure of importin compared to its unbound conformation, it can be safely be assumed that the changes we observed are due to any of the reasons above.

As further evidence of the binding, the thermal denaturation midpoint of the potential complex should increase when compared with that of either isolated Impα3 or ΔImpα3 [30]. Thermal denaturations followed by changes in the ellipticity at 222 nm in far-UV CD experiments showed: (i) a change in the sigmoidal shape of the thermal denaturations of the complex and those of the isolated importin species; and (ii) an increase in the thermal denaturation midpoints of the complexes, when compared to those obtained in the denaturations of the isolated importin species (Figure 4C). For instance, in the case of ΔImpα3 there was an increase in the thermal denaturation midpoint of almost 10 °C (44 °C versus 53 °C, although the large baselines observed in the unfolded and native sections of the thermal denaturation curves could hide the true value of the thermal denaturation midpoint). In the case of Impα3, the changes in the thermal denaturation midpoints, and in the shape of the thermal denaturation curves, were not as large as those of the complex with ΔImpα3, in an apparent agreement with the smaller changes observed in the steady state spectra of the complex and the addition one (Figure 4A,B): 52 °C for isolated Impα3 and 56 °C for Impα3 with the NLS-Myc.

Thus, taking together all these observations, we can conclude that there was binding between the peptide and any of the two importins. On the basis of these findings, our next step was to provide a quantitative measurement of such binding. At this stage it is important to indicate that, although peptide binding affinity and protein structural stabilization are intimately related, there is no direct correlation between the two observations; this means that a peptide exhibiting the same affinity for two different proteins does not necessarily induce the same stabilization effect in the thermal denaturation experiments. Therefore, protein stability increments are not useful to rank peptide binding affinities, and the increased stability observed upon thermal-denaturation (Figure 4C) may be the result of unspecific interactions between NLS-Myc and both importins.

Since we observed changes in the fluorescence spectrum upon binding of NLS-Myc to both Impα3 and ΔImpα3, we carried out titrations by keeping constant the concentration of each of the importin species and increasing the concentration of the peptide. The results indicate (Figure 3B,D) that, although there was a decrease of the fluorescence intensity upon addition of the peptide, the spectrum obtained did not follow the binding model we expected (see Equation (1) in the 4.2.2. Binding Fluorescence Experiments on NLS-Myc). This surprising result could be due to the fact that the *K*_d_ measured was not in the range of the concentrations explored by the fluorescence experiments, and it was smaller than the concentration of fixed importin species used (3 µM). Therefore, we used BLI as a technique which could explore a smaller concentration range.

**Figure 5:**
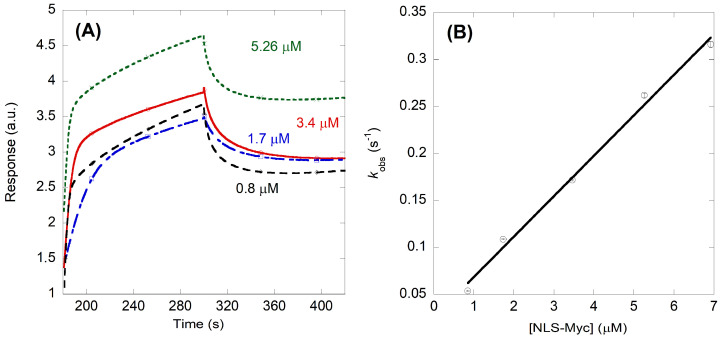
Binding of Impα3 to NLS-Myc as monitored by BLI: (**A**) sensorgrams at different concentrations of NLS-Myc; and (**B**) Pseudo-first order plot of the binding of NLS-Myc to Impα3. The errors bars, within the circles, are fitting errors to Equation (4). The straight line is the fitting to Equation (5). Experiments were carried out at 25 °C.

The results of BLI measurements for Impα3 (Figure 5A) also indicated binding of NLS-Myc to the biosensor-bound importin species, and they yielded a value of the *K*_d_ of 0.6 ± 0.2 µM, with *k*_on_ = 0.0430 ± 0.002 µM^−1^ s^−1^ and *k*_off_ = 0.025 ± 0.008 s^−1^ (Figure 5B).

**Figure 6:**
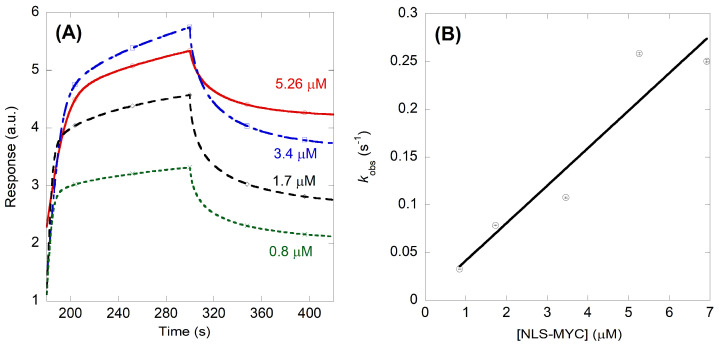
Binding of ΔImpα3 to NLS-Myc as monitored by BLI: (**A**) sensorgrams at different concentrations of NLS-Myc; and (**B**) pseudo-first order plot of the binding of the peptide to ΔImpα3. The errors bars, within the circles, are fitting errors to Equation (4). The straight line is the fitting to Equation (5). Experiments were carried out at 25 °C.

On the other hand, for ΔImpα3 (Figure 6A), the *K*_d_ was 0.06 ± 0.03 µM, with *k*_on_ = 0.039 ± 0.007 µM^−1^ s^−1^ and *k*_off_ = 0.002 ± 0.001 s^−1^ (Figure 6B). The low values of the *K*_d_ obtained from BLI measurements for both importin species would explain why we were not able to observe a titration from fluorescence (Figure 3B,D) at the concentration range explored with our Cary spectrofluorometer. Therefore, from a kinetic point of view, we can conclude that: (i) the association rate of NLS-Myc to both importin species was nearly the same, and in the order of 0.040 µM^−1^ s^−1^; and (ii) the higher affinity for ΔImpα3 (i.e., a smaller dissociation constant) was due to a much smaller (one order of magnitude lower) dissociation rate.

### 2.3. NLS-Myc Was Bound to the Major NLS Binding Site of Impα3

Docking simulations were performed to study at atomic detail the binding of the NLS-Myc peptide on the surface of Impα3. Thirteen fragments (named from F1 to F13) encompassing the whole peptide sequence were individually screened, as reported in Table 1. The most striking result we obtained is that the best docking pose in each simulation run was observed in the main binding site of Impα3 for cargo sequences. This included the docking poses of the fragments F6–F8, encompassing the central region of the NLS-Myc peptide. This result was not obvious due to the differences between the NLS of Myc compared to that of the Ran-binding protein, which is the crystallographic ligand in the structure of Impα3 used for the simulations [31], and especially because of the difference in their core NLS sequence (as detailed later in the Section 4.6.1. Structure of the Docking Receptor Impa3).

**Figure 7:**
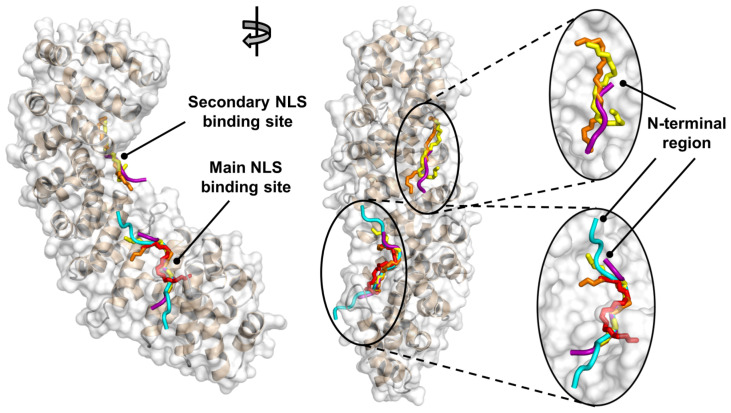
Simulated bound structures of NLS-Myc to Impα3/ΔImpα3. Docking poses (in stick representation) of fragments F6 (yellow), F7 (orange), and F8 (red) of NLS-Myc are compared with the crystallographic NLS (in tube representation) of the Ran-binding protein (cyan) bound to Impα3 (PDB entry: 5XZX [31]) and of Myc (purple) bound to karyopherin α from *Saccharomyces cerevisiae*, (PDP entry: 1EE4 [13]). (Left) Lateral view, highlighting the position of the NLS binding sites along the protein ARM units. (Center) Rotated longitudinal view. (Right) Insets showing details of the two binding sites. The sole –N–C^α^–C– backbone is shown for both the simulated and crystallographic NLS sequences, for clarity.

Figure 7 shows the predicted binding location of fragments F6, F7, and F8, which taken together encompass the canonical NLS of Myc (sequence: P^320^AAKRVKLD^328^ [9]). The calculated binding affinities towards Impα3 were, respectively, −6.9, −6.5, and −6.7 kcal/mol (Table 1). A comparison with the two crystallographic NLSs (PDB entries: 5XZX and 1EE4 [13,31]) confirms that these fragments occupy the main binding site of Impα3. The agreement in the superposition of the main chain of the fragments and the two NLSs was very good, and the empirical nature of the docking score function and intrinsic stochasticity of the search process contribute to produce small differences.

It is interesting to point out that the docking poses of these three fragments were also successful in approximately reproducing the correct shift by two residues in the position of F7 compared to F6, and of F8 compared to F7. Furthermore, the simulations also reproduced the correct fragment orientation (i.e., the three fragments had in all cases the C-terminal region pointing roughly downwards in Figure 7, precisely like the crystallographic NLSs [13,31]). In our simulations we also observed some docking poses possessing the non-canonical (i.e., opposite) orientation, although the calculated affinity was less favorable (by 0.5 kcal/mol or more). This adaptability could be a feature of Myc, which is unique in the fact that peptides corresponding to its canonical NLS with both forward and reverse sequence can bind to the importin α binding site, as demonstrated through in vitro cell assays [32].

A few docking poses were also found in a sole other location, corresponding to the secondary binding site for the NLS of Myc that is uniquely reported on karyopherin α from *S. cerevisiae* (PDB entry: 1EE4 [13]). These docking poses were slightly displaced compared to the position of the NLS in crystallography (Figure 7), due to the different topography of such protein crevice in karyopherin α compared to our docking target, Impα3. The calculated affinities for the binding of fragments to these locations were less favorable by 0.5–1.0 kcal/mol, compared to the binding energies found in the main binding site. This result, together with (i) the close proximity of the secondary site to the main binding pocket of Impα3, and (ii) the complete solvent accessibility of both sites, suggests that such secondary binding site may be significantly loaded only under the specific conditions experienced in crystallography (e.g., excess of ligand concentration, damped dynamics of the host, and high dehydration of the binding complex).

We stress that, in our simulations, all the other fragments F1–F5 and F9–F13 were also observed to bind in the main binding site of Impα3 for cargo sequences. The distribution of their binding modes was less defined (i.e., it corresponded to a cluster of conformations similar to each other, rather than a single distinct one), and their positions sometimes overlapped with the anchoring location of the core region of the NLS. This may seem counterintuitive at first, as it could have been expected that they would rather flank the position of fragments F6–F8 on each respective side, roughly superimposing to the backbone of their crystallographic NLS counterparts. We have observed the same behavior in other similar cases [18,33], and raised the hypothesis that fragments adjacent to the core NLS binding motif having a good affinity for the same protein anchoring site could be convenient in the first steps of the recognition process or contribute to hampering the dissociation of the NLS when bound, thus favoring/disfavoring the kinetics of association/dissociation. Nevertheless, the region encompassing fragments F6–F8 is the sole expected to accommodate in that position at the end of the binding reaction, due to its slightly more favorable affinity for that protein pocket compared to the other fragments.

## 3. Discussion

Myc is a nuclear transcription factor, which is a central hub inside the nucleus modulating signals from several pathways involved in direct gene expression programs. Its NLS has been identified [9], and the X-ray structure of the complex with yeast importin α has been solved by X-ray [13]. Although (i) a shorter version of the NLS of Myc, comprising residues Pro320-Asp328, has been used in competition experiments with other NLSs; and (ii) fluorescence depolarization assays with a peptide containing a larger number of flanking residues and the green fluorescence protein (GFP) have yielded a *K*_d_ with ΔImpα of *S. cerevisiae* [34] of 6 ± 3 nM, no biophysical and spectroscopic studies describing its interaction with human Impα3 and ΔImpα3 in vitro have been undertaken. Furthermore, no studies describing the conformational preferences of the isolated NLS of Myc in solution had been carried out so far, to the best of our knowledge.

This investigation is part of a more general endeavor in which we are using Impα3, with and without its IBB domain, as a model karyopherin to dissect out the binding of the NLSs of well-folded proteins and IDPs. In fact, association of a cargo protein to a nuclear transporter is regulated by a complex interplay of interactions among (i) the binding sites in the ARM domain (which are two not only to host bipartite localization signals, but sometimes even shorter monopartite sequences, as it happens for Myc [13]); (ii) the IBB domain, whose self-binding to the ARM domain has an auto-inhibitory effect that acts as a regulatory switch [19,20]; and (iii) the NLSs of the binder protein (which in many cases can have multiple localization signals). Our studies may pave the way to future and more complex ones, because numerous different cargoes compete in vivo for the binding, and selectively inhibiting nuclear translocation of disease-related proteins can be exploited for therapeutic purposes [23]. These studies are pioneering for IDPs, whose translocation process is still obscure in many aspects. However, they also increase our understanding of the same mechanism for well-folded proteins, because NLSs share many aspects with disordered proteins (e.g., they are highly charged and have solvent-exposed residues). Thus, understanding the conformational preferences of NLSs in solution, both in isolation and considered within the intact protein they belong to, is particularly interesting.

One of the results of the present work is that the isolated NLS-Myc peptide is disordered in solution, as indicated by CD and NMR (Figure 2). This region was initially predicted to adopt an α-helix-like conformation [9], although more recently a disordered predictor (PONDR) indicated that it should be mainly disordered [35], and X-ray crystallographic studies have also showed that most of its structure in the bound state could not be solved [13]. Conversely, the CD results (Figure 4A,B) suggest that, when NLS-Myc was bound to either Impα3 or ΔImpα3, the peptide acquired a folded conformation. The X-ray crystal structure with the *S. cerevisiae* importin α indicates that the canonical NLS Pro320–Asp328 attained, when bound, an extended structure with a bending comprising residues Pro320–Ala321 [13,35]; therefore, the changes observed in the CD experiments in our work must be due either to: (i) conformational preferences of the flanking residues (i.e., residues Ala310–Tyr319 and Ser329–Asn339) when bound to importin; or (ii) changes occurring upon binding in the environment of some aromatic residues, which also absorb in the far-UV CD region in that wavelength range, around either Tyr319 in NLS-Myc or several possible Tyr/His/Trp residues in the importin species [36,37,38,39]. On the other hand, the simulation results clearly point out to a well-defined anchoring region in the main NLS binding site of Impα3/ΔImpα3, corresponding in fact to residues Pro320–Ser329.

Human c-Myc is an IDP with a largely disordered structure (Figure 1A) [35]. Previous studies on other NLSs of IDPs [18,19,20] or, alternatively, of folded proteins [15,16,33,40] have suggested an inhibitory action of the IBB of Impα3: this domain hampers the anchoring of the NLS of the corresponding cargo protein into the major NLS-binding region of Impα3 [12,41]. In this respect, from BLI measurements, we have observed an affinity one-order of magnitude higher in the binding of NLS-Myc towards ΔImpα3 compared to Impα3, in agreement with the findings obtained for other NLSs assayed before [18,19,20,33]. The main difference in the binding between the two importin species is due to the presence of the 60-residue-long IBB (Figure 1B), which is competing with the NLS for the NLS-anchoring region. Such a variation between the affinities of either Impα3 or ΔImpα3 for the same NLS was not as large as the one we detected for other NLSs explored [18,19,20,33]; furthermore, the values of the affinities for NLS-Myc of either Impα3 or ΔImpα3 were much more favorable (i.e., had a smaller dissociation constant) than those measured for other NLSs [18,19,20,33]. Thus, it seems that the sequence of NLS-Myc is much more optimized for binding to Impα3/ΔImpα3 than those of the other proteins we tested. However, the value of the *K*_d_ for human ΔImpα3 obtained here was larger (60 nM) than that measured by using a peptide attached to GFP (6 nM) towards yeast ΔImpα [34]; the difference can be due to the different measurement conditions, or to the specificity that some importins have for their carriers [42].

From a kinetic point of view, we observed that the binding of NLS-Myc for both importin species occurred at nearly the same speed during association, but dissociation from ΔImpα3 was much slower. This slower dissociation rate (*k*_off_) is the reason of the higher affinity (smaller *K*_d_) of the NLS-Myc peptide towards ΔImpα3. We have not observed such small dissociation rates in other NLSs from other proteins for which the kinetic rates have been measured [33], which suggests some difference in the release mechanism (e.g., the presence of a metastable state, or a kinetic barrier). Our docking simulations showed that (i) the residues flanking the core NLS of Myc have a slightly lower affinity for the same anchoring location of the core region itself; and (ii) a secondary binding site for the NLS of Myc may also exist for Impα3/ΔImpα3 in close proximity to the main binding site, although its lower affinity with respect to the latter suggests it should not be significantly populated under ordinary conditions. Since in both cases the energetic differences calculated are quite small (within 1 kcal/mol), these findings may contribute to explaining the existence of mechanisms that could slow down the dissociation of NLS-Myc from Impα3. On the other hand, the fact that our docking simulations indicate that NLS-Myc occupies the major groove in Impα3 raises the intriguing possibility that NLS-Myc (or another similar peptide) could be used as a kinetic inhibitor of Impα3 when bound to other different cargos.

In conclusion, our results contribute towards clarifying the structural properties of a key region of Myc, which is relevant because of the importance of such nuclear transcription factors in many mechanisms leading to cancer. Furthermore, the findings here reported shed further light on the binding properties of Impα3, a versatile and highly conserved transporter of cargoes within the karyopherin family.

## 4. Materials and Methods

### 4.1. Materials

#### 4.1.1. Chemicals

Imidazole, Trizma base, DNase, SIGMAFAST protease tablets, NaCl, Ni^2+^-resin and ultra-pure dioxane were purchased from Sigma (Madrid, Spain). The β-mercaptoethanol was from BioRad (Madrid, Spain). Ampicillin and isoproyl-β-D-1-thiogalactopyranoside were obtained from Apollo Scientific (Stockport, UK). The SDS protein marker (PAGEmark Tricolor) was from VWR (Barcelona, Spain). Amicon centrifugal devices with a molecular weight cut-off of 30 kDa were from Millipore (Barcelona, Spain). The rest of the used materials were of analytical grade. Water was deionized and purified on a Millipore system.

#### 4.1.2. Protein Expression and Purification

Impα3 and ΔImpα3 were purified as previously described [18,19,20]. Protein concentrations were determined by ultraviolet (UV) absorbance, employing an extinction coefficient at 280 nm estimated from the number of tyrosines and tryptophans in each of these proteins [43].

#### 4.1.3. Synthesis of NLS-Myc

The NLS-Myc peptide comprised the previously identified canonical NLS of Myc (from Pro320 to Asp328), flanked at both sides by the corresponding regions of native Myc. The sequence of NLS-Myc is: A^310^APPSTRKDYPAAKRVKLDSVRVLRQISNN^339^ (in the numbering of the intact Myc), with a molecular weight of 3351.89 Da. The peptide was produced by NZYtech (Lisbon, Portugal), with a purity larger than 95%. Its concentration was determined from the absorbance of the sole residue Tyr319 [43].

### 4.2. Fluorescence

#### 4.2.1. Steady-State Fluorescence

Fluorescence spectra were collected on a Cary Varian spectrofluorometer by Agilent (Santa Clara, CA, USA), interfaced with a Peltier unit. Following the standard protocols used in our laboratories, the samples were prepared the day before and left overnight at 5 °C; before experiments, samples were left for 1 h at 25 °C, and at that point experiments were acquired. A 1-cm-pathlength quartz cell by Hellma (Kruibeke, Belgium) was used. Concentration of both Impα3 and ΔImpα3 was 2 µM, and that of NLS-Myc was 20 µM. Samples containing either the isolated NLS-Myc, the two isolated importin species, or the corresponding mixtures (at the concentrations indicated above) were prepared. Experiments were performed with samples in 50 mM sodium phosphate buffer, at pH 7.0. Fluorescence experiments were repeated in triplicates with newly prepared samples. Variations of results among the repeated experiments were lower than 5%.

Protein or peptide samples were excited either at 280 or 295 nm (although NLS-Myc has a sole Tyr residue). The other experimental parameters used in the experiments have been described elsewhere [44]. Appropriate blank corrections were made in all spectra.

#### 4.2.2. Binding Fluorescence Experiments on NLS-Myc

For the titration between either Impα3 or ΔImpα3 with NLS-Myc, increasing amounts of the latter, in the concentration range 0–35 µM, were added to a solution with a fixed concentration of the corresponding importin species (3 µM). Experiments were carried out in buffer: 20 mM Tris buffer (pH 7.5) and 150 mM NaCl, at 25 °C. The samples were excited at 280 and 295 nm, and the rest of the experimental set-up was the same as in the steady-state fluorescence experiments. In all cases, the appropriate blank-corrections, matching the corresponding amounts of NLS-Myc, were subtracted. Spectra were corrected for inner-filter effects during fluorescence excitation [45]. Each titration (Impα3 with NLS-Myc, or ΔImpα3 with NLS-Myc) was repeated three times, using new samples for each experiment. In the three cases, the variations in the results were lower than 10%.

The samples were prepared the day before and left overnight at 5 °C; before the measurements, the samples were incubated for 1 h at 25 °C. The dissociation constant of the corresponding complex, *K*_d_, was calculated by fitting the binding isotherm constructed by plotting the observed fluorescence change as a function of importin species concentration to the general binding model, explicitly considering ligand depletion [46,47]:

(1) $$\begin{array}{c} F=F_{0}+\frac{\Delta F_{\max}}{2{\left[{\mathrm{Imp}\alpha 3}_{\mathrm{species}}\right]}_{T}}({\left[\mathrm{NLS}-\mathrm{Myc}\right]}_{T}+{\left[{\mathrm{Imp}\alpha 3}_{\mathrm{species}}\right]}_{T}+K_{d}\\ )\;\;\;\;\;\;-\sqrt{\left({\left({\left[\mathrm{NLS}-\mathrm{Myc}\right]}_{T}+{\left[{\mathrm{Imp}\alpha 3}_{\mathrm{species}}\right]}_{T}+K_{d}\right)}^{2}-4{\left[{\mathrm{Imp}\alpha 3}_{\mathrm{species}}\right]}_{T}{\left[\mathrm{NLS}-\mathrm{Myc}\right]}_{T}\right)} \end{array}$$

where *F* is the measured fluorescence at any particular concentration of NLS-Myc after subtraction of the blank with the same concentration of NLS-Myc; Δ*F*_max_ is the largest change in the fluorescence of NLS-Myc when all polypeptide molecules were forming the complex, compared to the fluorescence of the isolated chain; *F*_0_ is the fluorescence intensity when no NLS-Myc was added; [Impα3_species_]_T_ is the constant, total concentration (3 µM) of either Impα3 or ΔImpα3; and [NLS-Myc]_T_ is the concentration of the peptide, which was varied during the titration. Fitting to Equation (1) was carried out by using KaleidaGraph by Synergy software (Reading, PA, USA).

### 4.3. Circular Dichroism (CD)

Far-UV CD spectra were collected on a J810 spectropolarimeter by Jasco (Tokyo, Japan) with a thermostated cell holder and interfaced with a Peltier unit. The instrument was periodically calibrated with (+)-10-camphorsulfonic acid. A cell by Hellma (Kruibeke, Belgium) of path length 0.1 cm was used. All spectra were corrected by subtracting the corresponding baseline. Concentration of each polypeptide (either importin species, and the NLS-Myc peptide) and the buffers were the same used in the fluorescence experiments (Section 2.2).

Isothermal wavelength spectra of each isolated macromolecule and that of their complex were acquired as an average of 6 scans, at a scan speed of 50 nm/min, with a response time of 2 s and a bandwidth of 1 nm. Samples were prepared the day before and left overnight at 5 °C to allow them to equilibrate. Before starting the experiments, samples were further left for 1 h at 25 °C.

Thermal denaturations were also carried out with the samples containing either importin species together with NLS-Myc, and those containing only Impα3 or ΔImpα3. In all cases, denaturations were acquired with a thermal slope of 60 °C/h by collecting data every 0.2 °C, with a bandwidth of 1 nm, and with a response time of 8 s. The unfolding was assessed by observing the ellipticity at 222 nm. Thermal denaturation midpoints of the isolated importin species or of the corresponding complexes were determined as described elsewhere [44]. Thermal denaturations were used because, if there is binding, the thermal denaturation midpoint of the complex with NLS-Myc should increase when compared with that of either isolated Impα3 or ΔImpα3 [48].

### 4.4. Nuclear Magnetic Resonance (NMR) Spectroscopy

The NMR spectra were acquired at 10 °C on a Bruker Avance 500 MHz spectrometer (Bruker GmbH, Karlsruhe, Germany), equipped with a triple resonance probe and z-pulse field gradients. Spectra were processed with Bruker TopSpin 2.1 (Bruker GmbH, Karlsruhe, Germany). All NMR experiments with NLS-Myc were carried out in 100 mM sodium phosphate buffer (not corrected for isotope effects), at pH 7.0. Spectra were calibrated with 3-(trimethylsilyl) propionic acid-2,2,3,3-^2^H_4_-sodium salt (TSP), by considering pH-dependent changes of its chemical shifts; the probe temperature was calibrated with pure methanol [49].

#### 4.4.1. 1D-^1^H-NMR Spectra

A total of 64 scans were acquired with 16 K acquisition points for the homonuclear 1D-^1^H-NMR spectra of each isolated peptide at a concentration of 1.2 mM. The water signal was suppressed with the WATERGATE sequence [50]. The spectra were processed after zero-filling and apodization with an exponential window.

The spectra in the presence of 100% D_2_O in the buffer indicated above were acquired 5 min after dissolving an amount of NLS-Myc to yield a final concentration of 345 µM in the final volume of 500 µL. The conditions used for data acquisition were the same as described above (Figure S1).

#### 4.4.2. Translational Diffusion NMR (DOSY)

The concentration of NLS-Myc in DOSY experiments was the same used in the 2D-^1^H-NMR spectrum (1.8 mM of peptide), and 96 scans were acquired with the gradient strength varied linearly. Measurements of the translational self-diffusion coefficient, *D*, were performed with the pulsed-gradient spin-echo sequence in the presence of 100% D_2_O. Details on the experimental conditions and fitting of the resulting curves have been described elsewhere [44]. The gradient strength was varied in sixteen linear steps between 2% to 95% of the total power of the gradient coil. Gradient strength was calibrated by using the value of *D* for the residual proton water signal in a sample containing 100% D_2_O, in a 5-mm tube [51]. The length of the gradient was 2.5 ms; the time between two pulse gradients in the pulse sequence was 250 ms; and the recovery delay between the bipolar gradients was 100 µs. The methyl groups with a signal between 0.8 and 1.0 ppm were used for peak integration, for NLS-Myc (Figure S3). A final concentration of 1% of dioxane, which was assumed to have a hydrodynamic radius *R*_h_ = 2.12 Å [51], was added to the solution.

#### 4.4.3. 2D-^1^H-NMR Spectra

Two-dimensional spectra of NLS-Myc were acquired in each dimension in phase-sensitive mode by using the time-proportional phase incrementation technique [52] and a spectral width of 5500 Hz (11 ppm); the concentration of the peptide was the same used in the 1D-^1^H- NMR experiments. Standard TOCSY (with a mixing time of 80 ms) [53] and NOESY experiments (with a mixing time of 250 ms) [54] were performed by acquiring a data matrix size of 4096 × 512 points. The decoupling in the presence of scalar interactions (DIPSI) spin-lock sequence [55] was used in the TOCSY experiments with a relaxation time of 1 s. A total of 96 scans were acquired per increment in the first dimension, and the residual water signal was removed by using the WATERGATE sequence [50]. NOESY spectra were collected with 128 scans per increment in the first dimension, using again the WATERGATE sequence [50], and with a relaxation time of 1 s. Data were zero-filled, resolution-enhanced with a square sine-bell window function optimized in each spectrum, and baseline-corrected. The ^1^H resonances were assigned by standard sequential assignment processes [26]. The chemical shift values of H_α_ protons in random-coil regions were obtained from tabulated data, corrected by neighbor residue effects [26,27,28].

### 4.5. Biolayer Interferometry (BLI)

#### 4.5.1. Experimental Design of BLI Experiments

The association (*k*_on_) and dissociation (*k*_off_) rate constants of the binding of NLS-Myc peptide to Impα3 or ΔImpα3 were determined by using a BLItz system (Pall ForteBio, Barcelona, Spain) [56]. The buffer used in the experiments was that recommended by the manufacturer. Since Impα3 and ΔImpα3 had a His-tag, they were immobilized on His-tag biosensors (Pall ForteBio, Barcelona, Spain) at 0.5 µM. The peptide concentrations were in the range from 1 to 7 µM during the association step.

The general scheme of the protein-association/dissociation reactions probed in the BLItz system for NLS-Myc were: 30 s of acquisition of the initial baseline with the 10 × kinetics buffer (provided by the manufacturer); 120 s of loading for Impα3 or ΔImpα3 into the biosensor; 30 s of baseline acquisition with the 10 × kinetics buffer; 120 s of association of NLS-Myc to the biosensor (which had been previously loaded with the importin species); and 120 s of dissociation of NLS-Myc from the biosensor in the 10 × kinetics buffer.

#### 4.5.2. Fitting of the BLI Sensorgrams

Fittings of the BLI sensorgrams was carried out by using KaleidaGraph by Synergy software (Reading, PA, USA) [57]. The interferometry response (measured in response units, RU) during the association step, *R*(*t*), and the binding rate, d*R*(*t*)/d*t*, can be used to evaluate the kinetics of the formation of the NLS-Myc complex between Impα3/ΔImpα3 and NLS-Myc, according to:

(2) $$\frac{dR\left(t\right)}{dt}=k_{on}\left[\mathrm{NLS}-\mathrm{Myc}\right]\left(R_{max}-R\left(t\right)\right)-k_{off}R\left(t\right)$$

where *R*_max_ is proportional to the total concentration of biosensor-bound importin-species; and [NLS-Myc] represents the concentration of the peptide.

In Equation (2), *R*(*t*) is given by:

(3) $$R\left(t\right)=R_{eq}-R_{eq}e^{\left(-k_{obs}\;\left(t-t_{0}\right)\right)}$$

where *R*_eq_ is the steady-state (or equilibrium) response obtained at virtually infinite time, when dR(t)/dt = 0, and t_0_ = 180 s is the time at which the association step between biosensor-immobilized Impα3/ΔImpα3 and NLS-Myc in the solution started. We fitted the value of *R*(*t*) experimentally obtained at any condition to:

(4) $$R\left(t\right)=R_{eq}-R_{eq}e^{\left(-k_{obs}\;\left(t-180\right)\right)}+{R^{\prime }}_{eq}\left(t-180\right)$$

where *R*’_eq_ is a constant from the fitting. The observed rate constant, *k*_obs_, present in the sole exponential part of Equation (4) was concentration-dependent, and it was used for the pseudo-first-order plots, where the value of *k*_obs_ is given by:

(5) $$k_{obs}=k_{on}\;\left[\mathrm{NLS}-\mathrm{Myc}\right]+k_{off}$$

The dissociation process was always fitted to a single exponential, with the value of *R*(*t*) given by:

(6) $$R\left(t\right)=R_{1}\;e^{\left(-k_{off}\left(t-t_{0}\right)\right)}$$

where *t*_0_ = 300 s is the time at which the dissociation of the peptide from the biosensor-bound Impα3/ΔImpα3 started in our experimental set-up, and *R*_1_ is the response level when dissociation starts.

### 4.6. Molecular Docking

#### 4.6.1. Structure of the Docking Receptor Impα3

The structure of Impα3 was extracted from the crystallographic complex of the protein from Homo sapiens with the NLS of the Ran-binding protein (PDB entry: 5XZX [31]) bound in the main binding site for cargoes. In this crystallographic complex, the core NLS is K^52^REK^55^ and, therefore, it contains the protein residue Glu54. This residue is negatively charged and influences the arrangement of the side chains of the two nearby Lys residues, ultimately affecting the local details of the shape of the protein pocket where the core NLS is bound. In contrast, the core NLS of our NLS-Myc peptide is K^323^RVK^326^ and contains in the variable X position the residue Val325, which has a shorter, apolar side chain.

The simulated structures were also compared with the crystallographic complex between karyopherin α from *Saccharomyces cerevisiae* and the NLS of Myc (PDB entry: 1EE4 [13]), in which the host protein has a relatively high homology with our docking target. In fact, sequence identity between 5XZX and 1EE4 is 48%, which increases to 64% when amino acids having similar chemical features are included. In the 1EE4 complex, the guest is the canonical NLS of Myc (sequence: P^320^AAKRVKLD^328^ [9]), which was bound to the main NLS-binding site of the protein. In addition, a nearby secondary binding site is also evident in this complex, although in such site the position of the sole core NLS K^323^RVK^326^ was resolved [13].

#### 4.6.2. Binding of the Docking Guest NLS-Myc

The entire sequence A^310^APPSTRKDYPAAKRVKLDSVRVLRQISNN^339^ of NLS-Myc was screened by considering consecutive shorter fragments of six residues, following a protocol we have introduced to model in an exhaustive way the binding of relatively long peptide/protein sequences to their molecular targets [58,59,60,61]. Each of the fragments was shifted with respect to the previous one by two residues, up to include the whole sequence of the NLS-Myc, for a total of 13 fragments (A^310^APPST^315^, P^312^PSTRK^317^, …, R^334^QISNN^339^). The amino acids truncated at both the N- and C-terminal ends (or at either end, for the first and last residue) were capped by adding a methyl moiety, to avoid artifacts that would result from the introduction of a fictitious charged group.

The docking simulations were carried out by using AutoDock Vina, version 1.1.2 [62]. The procedure described above overcame the computational bottleneck due to an exceedingly large number of degrees of freedom of the docking guest; in fact, the number of rotatable bonds in the fragments were on average n = 26 ± 3, below the maximum (n ≤ 32) recommended for a reliable use of such docking engine [62]. The simulations were performed by carrying out a blind search within the whole protein volume, using default program settings. The search region centered on Impα3 had a size of 50 Å × 90 Å × 90 Å.

## Figures and Tables

**Table 1:** Screening of the binding of NLS-Myc fragments to Impα3.

Fragment	Sequence	Number of Rotatable Bonds	Binding Affinity (kcal/mol)
F1	A^310^APPST^315^	20	−6.3
F2	P^312^PSTRK^317^	26	−6.5
F3	S^314^TRKDY^319^	30	−6.5
F4	R^316^KDYPA^321^	28	−5.9
F5	D^318^YPAAK^323^	24	−6.4
F6	P^320^AAKRV^325^	21	−6.9
F7	A^322^KRVKL^327^	29	−6.5
F8	R^324^VKLDS^329^	28	−6.7
F9	K^326^LDSVR^331^	28	−6.3
F10	D^328^SVRVL^333^	24	−6.6
F11	V^330^RVLRQ^335^	27	−6.7
F12	V^332^LRQIS^337^	26	−6.4
F13	R^334^QISNN^339^	27	−6.7

## Data Availability

Data are available from the corresponding authors upon reasonable request.

## Supplementary Materials

The following supporting information can be downloaded at: https://www.mdpi.com/article/10.3390/ijms232315333/s1.

## Abbreviations

BLI	biolayer interferometry
CD	circular dichroism
DIPSI	decoupling in the presence of scalar interactions
DOSY	diffusion ordered spectroscopy
GFP	green fluorescent protein
IBB	importin β-binding domain
Impα3	human importin α3 isoform (residues 1–521)
ΔImpα3	IBB-depleted species of importin α3 (residues 64–521 of the intact protein)
NLS	nuclear localization sequence
NLS-Myc	peptide comprising the canonical nuclear localization sequence of Myc protein (residues 310–337 of the intact protein)
NMR	nuclear magnetic resonance
NOESY	nuclear Overhauser effect spectroscopy
NPC	nuclear pore complex
PDB	Protein Data Bank
TOCSY	total correlation spectroscopy
TSP	3-(trimethylsilyl) propionic acid-2,2,3,3-^2^H_4_-sodium salt
UV	ultraviolet
