# Supplementary material for: Deciphering the Binding of the Nuclear Localization Sequence of Myc Protein to the Nuclear Carrier Importin α3
Source: Int J Mol Sci. 2022 Dec 5;23(23):15333. doi: 10.3390/ijms232315333 (PMC9739371; doi:10.3390/ijms232315333)

Supplementary Material

Deciphering the binding of the nuclear localization sequence of Myc protein to the nuclear carrier Importin α3

Bruno Rizzuti, Juan L. Iovanna and José L Neira

SUPPPORTING TABLE

Table S1: Chemical shifts (δ, ppm from TSP) of NLS-Myc in aqueous solution (pH 7.0, 10 ºC)[a]

	NH	Hα	Hβ2	Hβ3	Hγ2	Hγ3	Hδ2	Hδ3	Hε	Hζ
Ala310		4.11 (-0.20)	1.60 (Me)							
Ala311	8.71	4.64 (-0.04)	1.37 (Me)							
Pro312		4.42 (-0.31)	1.99		2.37		3.77; 3.54			
Pro313		4.37 (-0.11)	2.04		2.37		3.77; 3.54			
Ser314	8.55	4.47 (-0.04)	3.87							
Thr315	8.40	4.37 (-0.01)	4.21		1.22 (Me)					
Arg316	8.47	4.34 (-0.02)	1.79		1.59					
Lys317*		4.31 (0.08)	1.75							
Asp318	8.35	4.59 (0.05)	2.59							
Tyr319	8.17	4.79 (-0.09)	3.05;2.86				7.16 (7.12 (cis))		6.83 (6.83 (cis))	
Pro320		4.45 (0.08)	2.05; 1.92		2.37		3.86; 3.67			
Ala321	8.32	4.26 (0.07)	1.36 (Me)							
Ala322	8.35	4.33 (0.007)	1.36 (Me)							
Lys323*	8.52	4.34 (0.06)	1.70		1.30					
Arg324	8.38	4.34 (-0.02)	1.82		1.57					
Val325*	8.28	4.09 (0.03)	2.03		0.92 (Me)					

Lys326	8.45	4.36 (0.03)	1.62	1.34	
Leu327	8.49	4.32 (-0.02)	1.81	1.62	0.90 (Me)
Asp328	8.50	4.61 (-0.04)	2.71		
Ser329	8.29	4.40 (-0.06)	3.88		
Val330	8.17	4.09 (-0.01)	2.10	0.93 (Me)	
Arg331	8.43	4.32 (-0.04)	1.80	1.62	
Val332*	8.28	4.09 (0.02)	2.03	0.92 (Me)	
Leu333	8.43	4.31 (-0.04)	1.80	1.69	0.85 (Me)
Arg334	8.38	4.34 (0.03)	1.82	1.57	
Gln335	8.54	4.46 (0.09)	2.03	2.68; 2.41	
Ile336	8.32	4.18 (-0.02)	1.87; 0.92 (Me)	1.48; 1.22	0.92 (Me)
Ser337	8.48	4.51 (0.03)	3.87		
Asn338	8.14	4.48 (-0.24)	2.79; 2.69		
Asn339	8.62	4.80 (0.12)	2.87, 2.75		

[a] The (*) indicates those residues whose resonances could not be unambiguously assigned. For the H_α proton column, the values within parenthesis are the conformational shifts (δ_{res}-δ_{rc}). The random-coil values for the sequence were obtained from: https://spin.niddk.nih.gov/bax/nmrserver/Poulsen_rc_CS/).

Figure S1. **1D ^{1}H-NMR spectrum of NLS-Myc dissolved in D_2O**. The amide region (top) and the methyl region (bottom) of a 1D ^{1}H-NMR spectrum after dissolving in D_2O. The signals appearing in the amide region at 6.81 and 7.16 ppm, respectively, correspond to the aromatic protons of Tyr319.

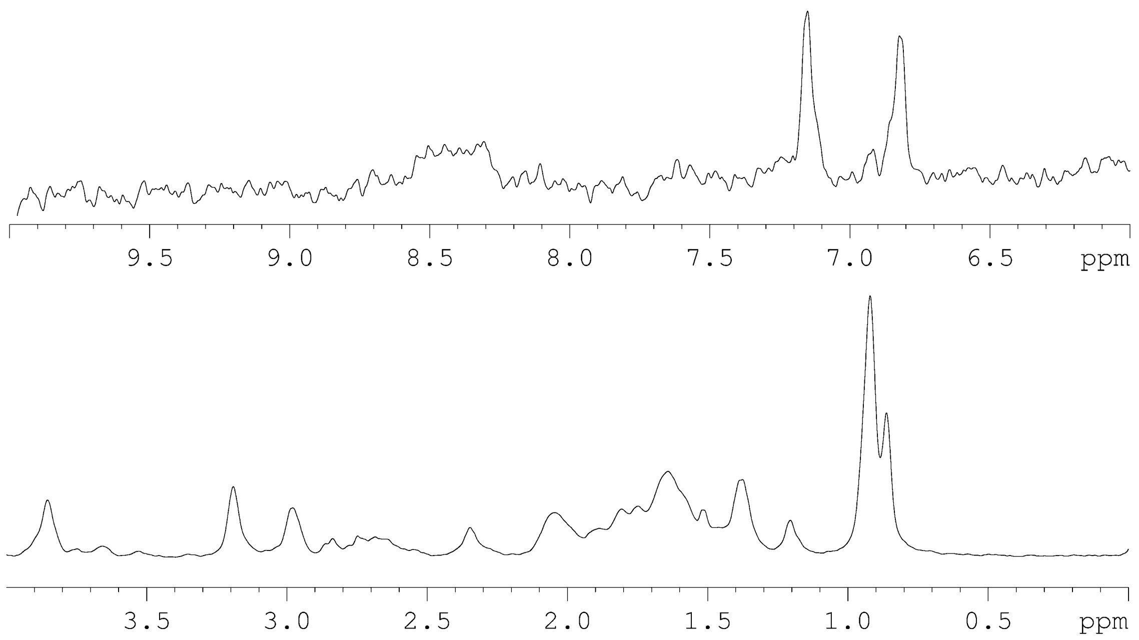

Figure S2. **Summary of NMR data for NLS-Myc peptide in aqueous solution.** NOEs are classified into strong, medium or weak according to the height of the bar underneath the sequence; signal intensity was judged by visual inspection from the NOESY experiments with 225 ms of mixing time. The corresponding H_α NOEs with the following H_δ of a proline residue are indicated by an open bar in the row corresponding to the $\alpha N(i, i+1)$ contacts. The dotted lines indicate NOE contacts which could not be unambiguously assigned due to signal overlapping or ambiguous assignment of the protons.

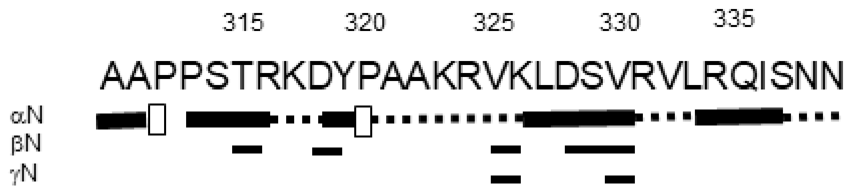

Figure S3. **DOSY NMR results**. Exponential fitting curve of the intensity of the methyl peak of the NLS-myc spectra as the square of the strenght of the gradient was increased.

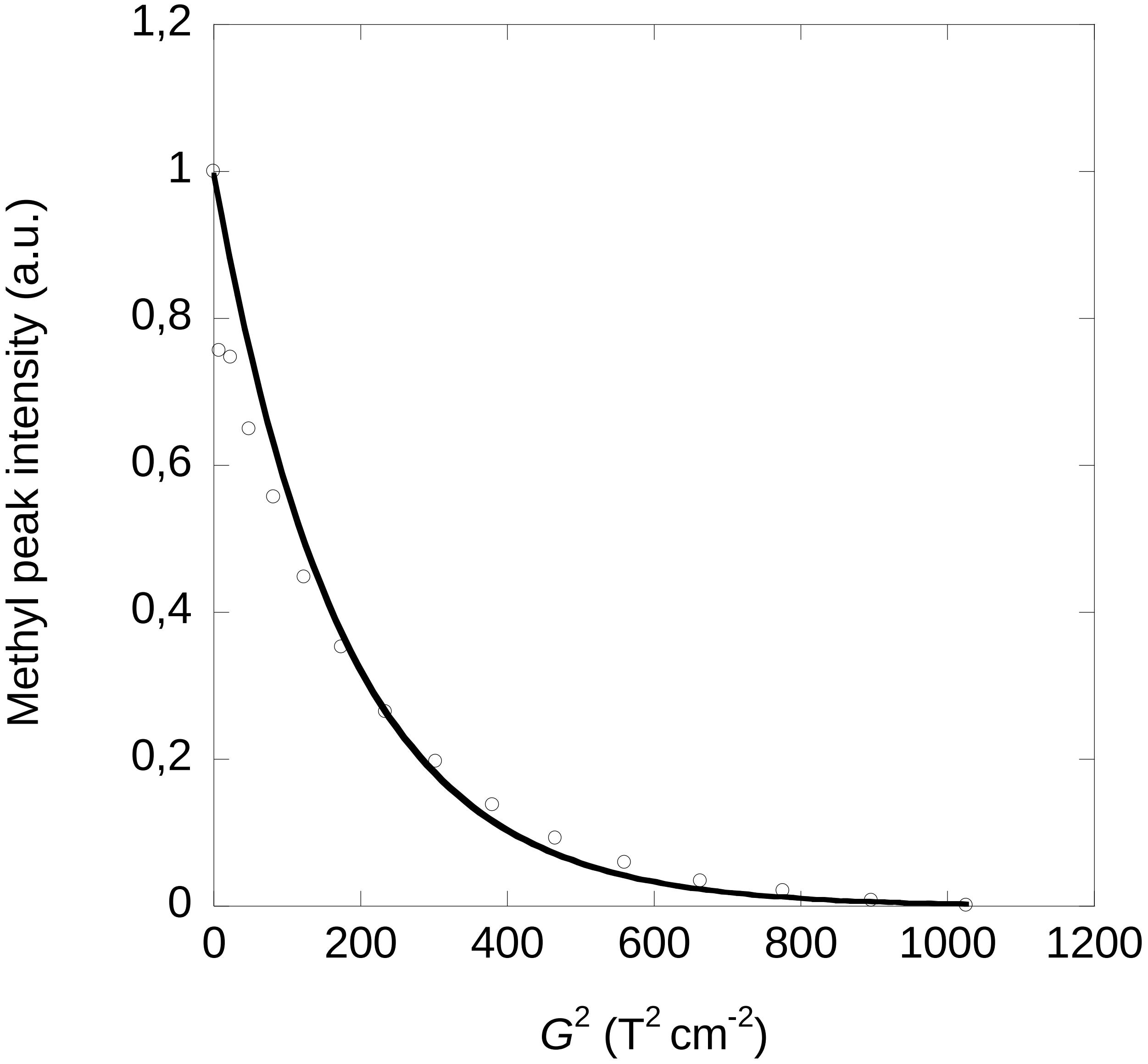

Supplement: Supplementary file 1 [file ijms-23-15333-s001.zip › ijms-2048581-supplementary.pdf]
